# Technical inefficiency of Dutch vegetable farms: Specific-input analyses

**DOI:** 10.1371/journal.pone.0250494

**Published:** 2021-04-23

**Authors:** Emmanuel Ahovi, Kevin Schneider, Alfons Oude Lansink

**Affiliations:** Business Economics Group, Wageningen University and Research, Wageningen, Netherlands; Shenzhen University, CHINA

## Abstract

Differences in technical efficiency across farms are one of the major factors explaining differences in farm survival and growth and changes in farm industry structure. This study employs Data Envelopment Analysis (DEA) to compute technical inefficiency scores for output, energy, materials, pesticides and fertiliser of a sample of Dutch indoor vegetable farms within the period 2006–2016. A bootstrap truncated regression model is used to determine statistical associations between producer-specific characteristics and technical inefficiency scores for the specified inputs. For the sample of indoor growers, the average technical inefficiency was about 14% for energy, 23% for materials, 24% for pesticides and 22% for fertilisers. The bootstrap truncated regression suggested that the degree of specialisation exerts adverse effects on the technical inefficiency of variable inputs. While age, short-term, long-term debt and subsidy were statistically significant, the coefficients were not economically significant. Building the capacity of farmers to reduce input inefficiency will enable farmers to be competitive and reduce the adverse effects of input overuse on the environment.

## Introduction

The horticulture sector comprising greenhouse and open ground production of vegetables and flower is the highest value sector in Dutch agriculture, followed by grassland-based livestock keeping [1]. In 2017–2019, vegetables and horticultural products contributed 39% of the total agricultural output [2].

Since the 1950s, Dutch horticulture has followed a model of increased intensification with increasing inputs of fertilisers, pesticides and energy. To consolidate and strengthen the Dutch position as the second agricultural exporter in the world, the process is continuing. Dutch horticulture is one of the most intensive production systems globally, with high output levels using the latest technologies [3, 4]. Therefore, it is not surprising that the average use of inputs such as fertilisers and pesticides per unit of land area is high [5]. Such intense farming systems may improve the utilisation of resources; they, however, may have negative impacts on biodiversity, greenhouse gas (GHG) emissions and air and water pollution, among others [4].

Although the Dutch government in recent decades has committed to sustainable agriculture, several studies [6–8] have reported considerable technical inefficiencies in Dutch agriculture. Only a few [8, 9] have focused on the horticulture sector [9, 10] studied the efficiency of CO_2_ emissions and energy focused on glasshouse horticulture but excluding insights into the efficiency of other inputs such as fertilisers, materials and pesticides, which also occupy an important place in Dutch horticulture. Other studies that focussed on vegetable production analysed overall technical efficiencies without references to specific inputs [11–13]. In turn, these studies recommended equi-proportionate reduction of all input. Arguably, the different inputs are managed differently by growers. Hence, inefficiencies related to specific inputs in horticulture need to be analysed to reflect their individual impact on the environment and the competitiveness of the horticulture industry.

Estimating inefficiency levels and drivers of inefficiency for specific inputs employed in the production process provides further insight for farm managers to improve their use of these inputs enabling better decision making. Information on input-specific inefficiency levels is also useful for policymakers in terms of specifying the policy instruments that promote greater levels of efficiency of inputs use and assessing the effectiveness of policies that have already been implemented [14].

As input inefficiencies are directly linked to the decisions of farm managers, it is worth investigating farm characteristics that could explain inefficiency differences between farms. Many studies have explained differences in technical inefficiency scores by farm characteristics such as age, years of farm managerial experience, received subsidies and capital structure [15–18]. Exploring determinants of inefficiency in relation to specific inputs is important to improve the performance of horticulture. Additionally, identifying such determinants of technical inefficiencies in Dutch vegetable production can be beneficial to other European countries and other countries with similar environmental and climatic factors.

This study aims to measure input-specific technical inefficiency of Dutch specialist indoor vegetable farms and identify farm-specific characteristics associated with inefficiency. This study makes two distinct contributions to the literature. First, we measure the technical inefficiency scores separately for energy, materials, pesticides and fertilisers for specialist indoor vegetable growers. This allows for more refined insight into the potential for reducing the use of specific inputs. Secondly, this study reveals farm characteristics that explain differences in the inefficiency of each input across farms. To achieve these, our study adopts a two-stage approach. First, data envelopment analysis (DEA) analysis is employed to measure technical inefficiency scores of the specified inputs used in the production of vegetables in the Netherlands. Second, a bootstrap truncated regression model is used to identify farm characteristics associated with technical inefficiency.

## Methodology

### Directional distance function

Consider for instance *i* = (1,…, *N*) decision making units (DMUs) which use *F* fixed inputs ${(x}_{i}^{f})$ and *S* variable inputs $x=(x_{1},x_{2}...,xs)\in R_{+}^{S}$ to produce a single output $y\in R_{+}$. A production technology can be fully characterised by the input requirement set

$$\text{T}(\text{y})=\left\{(x,y):\;x\;\right.\text{can}\;\text{produce}\;y,\text{given}\left.\;{\text{x}}^{f}\right)$$ (1)

The production technology *T(y)* can also be expressed non-parametrically as

$$T(y)=\left\{\left(x,x^{f}\right):Y^{\prime }\lambda \cdot \geq \cdot y_{i},X^{\prime }\lambda \leq x_{i},X^{f^{\prime }}\lambda \leq x_{i}^{f},I^{\prime }\lambda =1,\lambda \geq 0\right\},$$ (2)

where *Y* is the *(N* × *1)* vector of observed outputs; *y*_*i*_ is the observed output level of firm *i*; *X* is the *(N* × *S)* matrix of observed variable inputs; *x*_*i*_ is the vector of variable inputs used by firm *i*; *X*^*f*^ is the matrix *(N x F)* of observed fixed inputs; $x_{i}^{f}$ is the vector of fixed inputs used by firm *i*; λ is a *(N* × 1*)* vector of intensity variables (firm weights). The technology set is considered non-empty and convex. Under the production technology set, we assume outputs, fixed inputs and variable inputs are freely disposable, implying their reduction is not costly [19].

To estimate inefficiency scores for the output and inputs, a directional distance function was used. The directional distance function measures the amount that a given observation can be projected in the directions *g*_*y*_ and *g*_*x*_ until it reaches the frontier [20]. The output and input specific distances provide measures of inefficiency for all farmers. The directional distance function that aims at reducing variable inputs while simultaneously expanding output is expressed as:

$$\text{D}\left({\text{y}}_{\text{i}}\cdot x_{i}^{v},x_{i}^{f};g_{y},g_{x}\right)=\text{Max}\left(\beta :\left(y_{i},x_{i}^{v}\right)\in \text{T}\right)$$ (3)

In Eq (3), *g*_*x*_ and *g*_*y*_, refer to the directional vectors associated with variable inputs and outputs, respectively. *β* denotes technical inefficiency measuring the equal maximum contraction of all variable inputs and maximum expansion of all outputs. To account for differences in environmental conditions across time, Eq (3) was computed separately for every year. To obtain input specific inefficiency scores, a directional distance function was formulated to disaggregate technical inefficiency as follows:

$$\vec{D_{0}^{t}}\left(y_{i},\cdot x_{1_{i}},x_{2_{i}}\cdot x_{3_{i}},x_{4_{i}}:g_{y},g_{x_{1}},g_{x_{2}},g_{x_{3}},g_{x_{4}}\right)=\text{Sup}\left\{\left(\beta_{y},\beta_{x_{1}},\beta_{x_{2}},\beta_{x_{3}},\beta_{x_{4}}\right)\in \text{T}\left(\text{y}:x_{i}^{f}\right)\right\}$$ (4)

s.t.:

$$y_{i}+\beta_{y}g_{y}\leq \sum_{i}^{t}\lambda Y$$ (i)

$$x_{1_{i}}^{t}-\beta_{x_{1}}g_{x_{1}}\geq \sum_{i}^{t}{\lambda X}_{1}$$ (ii)

$$x_{2_{i}}^{t}-\beta_{x_{2}}g_{x_{2}}\geq \sum_{i}^{t}{\lambda X}_{2}$$ (iii)

$$x_{3_{i}}^{t}-\beta_{x_{3}}g_{x_{3}}\geq \sum_{i}^{t}{\lambda X}_{3}$$ (iv)

$$x_{4_{i}}^{t}-\beta_{x_{4}}g_{x_{4}}\geq \sum_{i}^{t}{\lambda X}_{4}$$ (v)

$$x_{i}^{f}\geq \sum_{i}^{t}{\lambda x}_{i}^{f}$$ (vi)

$$N1^{\prime }\lambda =1$$ (vii)

λ≥0

0<β≤1

Where *x*_1_, *x*_2_, *x*_3_ and *x*_4_ are scalar values of specific variable inputs used by farm *i*. $\beta_{x_{1}},\beta_{x_{2}},\beta_{x_{3}},\beta_{x_{4}}$ and *β*_*y*_ refer to the technical inefficiency concerning the separate variable inputs and output *y*, respectively. The inefficiency scores (*β*) take values between 0 and 1, except for *β*_*y*_ (can be greater than 1) where a value of 0 indicates no improvement potential. The vector of firm weights (λ) identifies the firms with the relevant reference points for a given observation, i.e. these are the firms that are located on the frontier. The constraint *N*1′ λ = 1 (with *N*1 being an *N* × 1 vector of ones) allows the sampled farmers to exhibit increasing, constant and decreasing return to scale (i.e. a variable return to scale (VRS) technology). To approximate the technology under constant returns to scale (CRS), we omitted the constraint *N*1′ λ = 1. To compute scale inefficiency, the difference between the VRS and CRS scores was computed.

### Second stage truncated bootstrap regression

To identify determinants of inefficiency, this study added a two-stage approach to account for nondiscretionary factors associated with the estimated inefficiency scores. Unfortunately, scores obtained from DEA are serially correlated by construction [21]. Thus, the use of Ordinary Least Squares would violate the independence assumption. To correct for this, Simar and Wilson [21] developed the single and the double truncated bootstrap procedures. This study used the single truncated bootstrap regression to estimate statistical associations of producer-specific characteristics and inefficiency scores [6, 22]. The Simar and Wilson [21] approach permits valid statistical inference to be made. Following Skevas, Oude Lansink and Stefanou [6], the single-bootstrap truncated regression model is defined as:

$$\beta_{k}=\delta Z+\varepsilon \geq 0$$ (5)

Where *β*_*k*_ is the estimated technical inefficiency score of input *k*. Following Schneider, Skevas, and Oude Lansink [23], inefficiency scores under the VRS assumption were used in the second stage as the VRS allows for a more flexible formulation of the production technology. *Ζ* denotes farm-specific characteristics (explanatory variables). *δ* denotes the vector of coefficients, and *ε* the error term. The distribution of the error term was assumed to be truncated normal, with zero mean, unknown variance, and left truncated at point 0- δΖ [21]. The maximum likelihood method was used to obtain estimates $\hat{\delta }$ and an estimate $\hat{\sigma_{\varepsilon }}$, where σ_ε_ denotes the standard deviation of the error term. Next, linear predictions of the inefficiency scores were generated based on the estimated coefficients and the producer-specific characteristics. For 2,000 iterations, errors were sampled out of a truncated distribution using the computed variance $\hat{\sigma_{\varepsilon }}$. Subsequently, truncated regression models were computed for all iterations by regressing the farm characteristics on the predicted inefficiency scores. Lastly, confidence intervals of the estimated coefficients were computed based on the empirical distribution to conclude on the statistical significance. As parameter estimates are only indicative of the direction of the relationship between inefficiency and farm characteristics, marginal effects were computed at the variables’ mean to enable interpretation of the association. Marginal effects measure the effect a unit increase/decrease in a particular explanatory variable has on the predicted inefficiency scores and estimated following the study of Singbo and Oude Lansink [24].

$$\frac{\partial E\left(\beta_{k}|Z,\beta_{k}>0\right)}{\partial z}=\left(\left(1-\frac{z^{\prime }\delta^{*}}{\sigma_{\varepsilon }^{*}}\times \frac{\phi \left(\frac{Z^{\prime }\delta^{*}}{\sigma_{\varepsilon }^{*}}\right)}{\Phi \left(\frac{z^{*}\delta^{*}}{\sigma_{\varepsilon }^{*}}\right)}-{\left[\frac{\phi \left(\frac{z^{\prime }\delta^{*}}{\sigma_{\varepsilon }^{*}}\right)}{\Phi \left(\frac{z^{*}\delta^{*}}{\sigma_{\varepsilon }^{*}}\right)}\right]}^{2}\right)\right)\delta^{*}$$ (6)

Where *β* is the estimated technical inefficiency score of input ***k***. *Z* is the mean of a particular explanatory variable. δ* is the bootstrapped coefficient for the explanatory variable, $\sigma_{\varepsilon }^{*}$ is the estimated variance of the error term. *ϕ*(·) is the standard normal distribution and Φ(·) is the standard normal cumulative distribution function.

### Data

Data on specialised vegetable farms for the period 2006–2016 were obtained from Wageningen Economic Research (WeCR). WeCR uses stratified sampling for specialist indoor Dutch vegetable farms, which are collected for the European Farm Accounting Data Network (FADN). The panel is unbalanced, and on average, farms stay in the sample for 6–7 years. After removing farms with missing information, the dataset used for estimation comprised 1090 observations from 222 indoor vegetable farms. Table 1 provides descriptive statistics of the variables in the dataset. For outputs and inputs (except land and labour), the available data contain information on revenues and expenses. Price indices for the specific output, fixed and variable inputs were obtained from Eurostat [25]. Implicit quantities of inputs and outputs are obtained by the ratio of monetary values and their respective price indices. For aggregate variables comprising several cost components, Tornqvist indices were constructed and used for the deflation [26]. One output and seven inputs (land, labour, capital, fertiliser, pesticide, energy and materials) were considered. Output consists of total revenue deflated with the price index for agricultural output using 2010 as a base year. Total revenue comprises of mainly vegetables, other horticultural products, cut flowers and turnips. Fixed inputs include capital, land and labour while variable inputs include energy, pesticides, fertilisers and materials. Capital comprises closing balance sheet values for machinery, buildings, glasshouses and installations deflated to 2010 prices using a Tornqvist price index.

**Table 1:** Descriptive statistics (Dutch indoor vegetable farms 2006–2016).

Variables	Dimension	Mean	SD*
Output	10000 Euros	180.00	266.74
Land	Hectares (ha)	6.84	7.68
Labour	10000 hours	2.70	3.60
Capital	10000 Euros	140.2	256.96
Fertilisers	10000 Euros	3.11	3.72
Pesticides	10000 Euros	2.16	2.92
Materials	10000 Euros	13.09	16.38
Energy	10000 Euros	47.46	69.25
*Farm Characteristics*			
Farmer’s age	10 years	4.90	0.85
Short term debt	-	0.12	0.68
Long term debt	-	0.48	0.66
Subsidies	10000 euros	2.48	5.70
HHI	[0,1]	0.76	0.19

* SD *denotes standard deviation*.

For the second stage bootstrap truncated regression, producer-specific characteristics such as capital structure is categorised into short- and long-term debt ratios. Short- and long-term debt were separated to investigate their differential association with technical inefficiencies. Table 2 shows descriptive statistics of the producer-specific characteristics that are available in the dataset. Age was measured as the age of the main holder. Short- and long-term debt were measured through the ratio of short- and long-term debt to total assets, respectively. The Herfindahl-Hirschman Index (HHI) was computed as a proxy for the farm specialisation [27, 28]. The index was computed by summing the squared revenue shares of arable crops, vegetables, flowers, cut-flowers, turnips and other horticultural and other revenue sources. HHI signifies a higher degree of specialisation as it approaches 1. Likewise, a value closer to 0 signifies a more diversified vegetable crop production. The mean value of the HHI index of 0.76 suggests Dutch indoor vegetable growers are highly specialised. The subsidy was measured as the total subsidy received from the government.

**Table 2:** Mean inefficiency scores for one output and four variable inputs.

	CRS	VRS	SI
Output	0.00	0.00	0.00
Energy	0.20	0.14	0.06
Materials	0.28	0.23	0.05
Pesticides	0.30	0.24	0.06
Fertilizers	0.29	0.22	0.07

Farmer’s age plays a dual role in explaining inefficiency. Older farmers are associated with lower inefficiency resulting from the experience gathered over the years in farming leading to better managerial skills [29]. On the other hand, younger farmers may be more motivated and willing to adopt new technologies and/or have a stronger educational background; hence they may be more efficient in using resources relative to older farmers [22]. Subsidies are expected to improve technical efficiency when they help farmers invest in new technologies; however, they may reduce farm performance when representing an income safety net. Until crops are harvested, the cost of inputs incurred during planting and growing creates negative cash flows [30]. This time gap between the time the inputs are used and the time of harvesting implies farmers’ need to have access to external funding. While the effect of short-term debt is unclear, long-term debt is expected to improve farm efficiency when invested in the farm business. It is expected that as a farm specialises in a single activity in total production, it gets in-depth knowledge over time and thus improves efficiency in that activity [31]. However, sometimes the benefits of diversification may outweigh the benefits of focusing on a single activity as farmers may diversify to reduce risk, especially for smallholder farmers [32].

## Results

### Input-specific technical inefficiencies

Table 2 presents the average technical inefficiency for output, energy, materials, pesticides and fertilisers under constant returns to scale CRS and VRS. Scale inefficiency (SI) -the difference in inefficiency under CRS and VRS for each input is also presented. The results indicate the maximum possible expansion in output and contraction in the use of variable inputs. Technical inefficiency scores of 0.00, 0.14, 0.23, 0.24 and 0.22 suggest that indoor growers have fully exploited the potential to expand output. However, the farmers could reduce energy, materials, pesticides and fertilisers respectively by 14%, 23%, 24% and 22% under the VRS assumption without changing output levels. A similar trend of inputs inefficiency scores could be observed for CRS. Inefficiency scores are quite similar for the various inputs except for energy. The sample of farmers analysed appears to use energy efficiently but tends to overutilise pesticides. The results also show that, on average, the farms are not operating at their optimal sizes as evidenced by their scale inefficiencies, i.e. improving the scale of production may lead to an additional saving of the use of inputs above the potential saving under VRS, of approximately 5–7%.

### Determinants of technical inefficiency

Table 3 presents the marginal effects of the inputs computed from the bootstrap truncated regression results. Output was excluded from the bootstrap truncated regression due to lack of variation in output inefficiency. Four explanatory variables were significant with energy, two were significant with materials, four were significant with pesticide and three were significant with fertiliser. For each input, the marginal effects indicate the direction of the statistical association interpreted by the value of their marginal effects. A negative marginal effect indicates that the parameter is associated with a reduction in inefficiency of the said input. Although statistically significant, most of the marginal effects are not economically relevant except for the degree of specialisation. For example, the marginal effect of a ten-year increase in age has a negligible reduction of approximately 0.07% on pesticide’s technical inefficiency. Also, for the ratio variables, unit increases in short- and long-term debt ratios are very large. In turn, the importance of these effects is also small.

**Table 3:** Marginal effects on the inefficiency of the separate inputs.

	Energy	Materials	Pesticides	Fertilizers
Age	4.90E-05	4.56E-04	-7.2E-04***	-4.5E-04
SD-ratio	0.106***	0.165***	0.143***	0.138***
LD-ratio	-0.022***	-0.004	-0.009	5.4E-04
Subsidies	-3.05E-07***	-1.4E-07	-2.0E-07***	-2.7E-07***
HHI	0.075***	0.311***	0.401***	0.423***

Note:

***P < 0.01;

**P < 0.05;

*P < 0.10.

## Discussion

According to the results, pesticides were the most technically inefficient variable input used by Dutch indoor vegetable farmers. This may suggest that the farmers are not keen on improving pesticide efficiency as it contributes the least to the total variable cost. As agricultural production activities are accompanied by a significant amount of risks, farmers may use pesticides as an insurance policy. Particularly for indoor cultivation, the high cost of investments and input cost results in increased production costs and consequently increased financial risks. Farmers at this point have a natural tendency to protect these investments through increased use of pesticides [33]. The production of vegetables attracts many different insects, often resulting in farmers overuse of pesticides as a countermeasure [34]. Oude Lansink and Silva [8] and Skevas and Oude Lansink [7] have also reported similarly high pesticide technical inefficiencies scores of 32.7% and 25% for Dutch arable farms for the period 1989–1992 and 2003–2007 respectively.

Energy had the lowest technical inefficiency score. This is supported by the findings of Oude Lansink and Silva [10], who reported a technical inefficiency score of 12% for the Dutch glasshouse industry for the period 1991–1995. Comparing to the levels in 1976–1995, energy use efficiency among Dutch greenhouse farms has improved over time [9]. Possibly, this could be related to the dependence of profits on energy costs for indoor growers [9]. Another factor could be the focus of policymakers to minimise energy use by the glasshouse industry. Golaszewski et al. [35] posit that a more energy-efficient agriculture will be increasingly demanded by food-chain partners and society and is also a necessity given competitiveness.

Technical inefficiency scores of materials and fertilisers also indicate an efficiency gap in the use of these resources. Given that land and other fixed production factors are limited, the efficiency of materials such as quality seeds, planting materials and fertiliser are important as they have a direct link with output volumes [36]. Materials have the second highest proportion of variable cost (Table 1) and thus, a reduction in the inefficiency of materials will contribute significantly to the economic health and competitiveness of the farms.

Results from the second stage bootstrap regression (Table 3) suggest that a higher degree of specialisation is associated with higher inefficiency of all inputs. Specifically, a one unit increase in the HHI was statistically associated with approximately 8%, 31%, 40% and 42% increase in the technical inefficiencies of energy, materials, pesticides and fertilisers, respectively. The result is in contrast with the findings of Singbo, Emvalomatis and Oude Lansink [37]. Dutch indoor vegetable growers are quite specialised, and it is possible that increased specialisation may lead to intensive use of inputs as farmers concentrate on a single activity, thus resulting in increased input inefficiency.

The farmers’ age, short-term and long-term debt ratios and subsidises were also found to have significant statistical associations with technical inefficiency of at least one of the inputs. One must, however, note that the economic importance of these marginal effects is not significant as substantial variations in these variables have only minimal effects on inefficiencies of the inputs. The direction of the statistical associations provides insights as it suggests precisely how changes in the explanatory variables may potentially impact inefficiency.

The negative marginal effect of age suggests that older growers may be statistically associated with lower inefficiency of pesticide use. This is supported by the study of Schneider, Skevas and Oude Lansink [23], who explained that older farmers gain experience over the years and hence make better managerial and investment decisions that result in improved farm performance.

Contrary to short term debt, the negative association between long-term debt ratio and inefficiency of energy may indicate investment in efficient innovations and technology resulting in improved energy and farm efficiency in general [29]. It may also be possible that farms with high long-term debt to total assets ratio may be creditworthy as they can provide sufficient collateral to lenders to get loans easily. However, it is worth noting that highly indebted farms may not have access to credit for working capital and therefore may not apply technological processes that improve efficiency [38]. Short-term debt is used to meet immediate and liquidity obligations of the farm such as the purchases of variable inputs and paying wages. Thus, undisciplined short-term borrowing might lead to inefficient use of variable inputs, resulting in higher input inefficiency, as shown by the positive statistical association.

This study suggests that the motivation for reducing inefficiency of the inputs (except materials) may be higher when the farmers receive subsidies. This is evidenced by the negative statistical association between subsidy and inefficiency. The impact of subsidy on farm’s efficiency can be double-sided. On one hand, it could lead to improvement in the efficiency of farms when subsidies are invested in improving the technology of production [29]. On the other hand, a subsidy can reduce farmers motivation to be efficient when they depend on it to a greater extent as an extra income or as insurance [39]. The negative relationship between subsidy and inefficiency scores in this study may indicate that the first effect dominates and that subsidies contribute to improvement in the production technology.

As inefficiency scores are a relative measure of performance, the number of DMUs can influence inefficiency scores. A low number of DMUs can result in a relatively higher number of efficient farms (farms with zero inefficiencies). The available dataset also limited our study on farm characteristics that could have potentially significant associations with technical inefficiency; that is, more farm and farmer-specific variables such as education level, family composition and the location of the farm may explain differences in technical inefficiency levels between farms.

## Conclusion

The study estimated the input-specific technical inefficiency among Dutch indoor vegetable farms for the period 2006–2016 and identified the sources of inefficiencies for the specified inputs.

Results from the study suggest considerable inefficiencies in the use of variable inputs among Dutch indoor vegetable farms. This implies that there is substantial scope for reducing inefficiency in the use of the inputs analysed. The results indicate that the sample of farmers, on average have fully exploited the potential to expand output. Meanwhile, they could reduce energy inefficiency by 14%, materials by 23%, pesticide by 24% and fertilisers by 22% while maintaining optimal output levels. Pesticides were identified to have the highest technical inefficiency score and energy the most efficiently used variable input. Results also show that scale improvements could lead to a 5–7% further reduction in the inputs’ use. The bootstrap truncated regression suggested that a higher degree of specialisation is significantly associated with a higher technical inefficiency of variable inputs. Age, short-term, long-term debt and subsidy were significant but devoid of economically significant effects.

The empirical results have implications for both policymakers and farm managers. The noticeable inefficiencies observed for the separate inputs necessitate policy actions to build farmers’ capacity to improve on the use efficiency of these inputs. Legislations should target the reduction of inputs such as energy, pesticides and fertilisers. This would consequently have positive impact in the environment through for example lower greenhouse gas emissions and lower runoff of minerals and pesticides, reducing at least costs. Alternatively, policies could be aimed at introducing levies on specific variable inputs when market incentives to improve efficiency fails.
